# ^68^Gallium-labelled fibroblast activation protein inhibitor uptake in joints: a single-center cohort analysis of 268 patients

**DOI:** 10.1007/s00259-025-07636-x

**Published:** 2025-11-20

**Authors:** Anna-Maria Spektor, Antonia van Genabith, Jorge Hoppner, Leon Walkenbach, Thomas Hielscher, Peter Kvacskay, Sarah Richter, Kiangenda Trésor Sungu-Winkler, Hans-Ulrich Kauczor, Hanns-Martin Lorenz, Mathias Schreckenberger, Jörg Distler, Uwe Haberkorn, Wolfgang Merkt, Manuel Röhrich

**Affiliations:** 1 Department of Nuclear Medicine, University Hospital Mainz, Mainz, Germany; 2 Department of Nuclear Medicine, University Hospital Heidelberg, Heidelberg, Germany; 3 Department of Biostatistics, German Cancer Research Center, Heidelberg, Germany; 4 Department of Internal Medicine V Hematology Oncology Rheumatology, University Hospital Heidelberg, Heidelberg, Germany; 5 Department of Diagnostic and Interventional Radiology, University Hospital Heidelberg, Heidelberg, Germany; 6 Translational Lung Research Center Heidelberg (TLRC), Member of the German Center for Lung Research DZL, Heidelberg, Germany; 7 Department of Rheumatology, University Hospital Düsseldorf, Düsseldorf, Germany; 8 Clinical Cooperation Unit Nuclear Medicine, German Cancer Research Center (DKFZ), Heidelberg, Germany

**Keywords:** FAPI, Fibroblasts, Osteoarthritis, PET, Joints

## Abstract

**Objective:**

Musculoskeletal diseases such as osteoarthritis (OA) are leading causes of pain, physical inactivity and disability worldwide. Synovial fibroblasts (SFs) expressing fibroblast activation protein (FAP) are crucial for OA progression. Positron emission tomography (PET) with ^68^Gallium-labelled FAP-inhibitors (^68^ Ga-FAPI) visualizes FAP-positive activated fibroblasts. Here, we systematically analyze FAPI-uptake respecting OA and joint degeneration in a cohort of 268 patients.

**Methods:**

^68^ Ga-FAPI-PET-scans of 268 oncological patients were analyzed for increased joint-associated FAPI-uptake, quantified by maximal, mean standardized uptake values (SUVmax/mean) and target to blood ratios (TBR) and compared with computed tomography-based OA-classification according to Kellgren and Lawrence. FAP-expression of SF from OA and rheumatoid arthritis (RA) (15 OA- and 26 RA-patients) were analyzed by single-cell cytometry by time of flight (cyTOF).

**Results:**

148 of 268 patients (55.2%) showed increased joint-associated FAPI-uptake (average SUVmax/mean of 3.25 ± 1.28/ 1.94 ± 0.70). The most frequent FAPI-positive joints were acromioclavicular and shoulder joints followed by sternoclavicular, lumbar facet and hip joints. FAPI-uptake was frequently asymmetric whereas OA-scoring displayed a symmetric allocation of degenerative changes suggesting discordance between FAPI-uptake and OA-scoring. Only in end-stage-OA (grade 4 according to Kellgren and Lawrence) FAPI-uptake was concordantly and significantly elevated. Abundant FAP-positive fibroblasts in end-stage-OA were confirmed by CyTOF**.**

**Conclusion:**

FAPI-uptake in joints is frequent and can occur in presence as well as in absence of joint degeneration. We suggest that FAPI-PET/CT complements conventional musculoskeletal imaging by providing information on synovial activity, the exact triggers of which remain to be elucidated.

**Supplementary Information:**

The online version contains supplementary material available at 10.1007/s00259-025-07636-x.

## Introduction

Osteoarthritis (OA) is one of the leading causes of pain, physical inactivity, disability and decreased quality of life [1–3] and is frequently encountered in the acromioclavicular, shoulder, knee and hip joints [4, 5]. It is a polyetiological, progressive, degenerative disease of the whole joint including its surrounding tissue such as muscles, subchondral bone, cartilage and ligaments resulting in joint deterioration [3, 6–8]. It is associated with synovial inflammation based on a dysregulated crosstalk between synovial fibroblasts (SFs) and macrophages [9]. SFs are the most frequent cell-type in synovia. In various diseases, SFs are plastic with subsets of various functions, including phenotypes that are tissue-destructive, recruit and activate immune cells [10–13]. SF-mediated synovitis contributes to structural deterioration of the cartilage, pain and swelling caused by increased nerve infiltration and synovitis-related effusion and thus to the onset and progression of OA [9, 14, 15]. OA is diagnosed based on clinical presentation, exclusion of differential diagnoses and supplemented by x-ray or magnetic resonance imaging (MRI) and can be classified radiomorphologically by the criteria of Kellgren and Lawrence (KL) [16]. Although the detection of early changes on a cellular level might improve the treatment of OA by early interventions, current imaging modalities neither allow a sensitive detection of early OA changes nor a prediction on disease progression [17].

The role of imaging with fibroblast activation protein inhibitors (FAPI) in oncological, fibrotic or inflammatory diseases is subject of current research. FAPI-uptake depicts active tissue remodeling which is present in chronically inflamed, fibrotic, degenerative or neoplastic tissue [18–22]. Positron emission tomography (PET) with computed tomography (CT) with FAPI (FAPI-PET/CT) was reportedly advantageous over standard modalities like MRI or CT for diagnosis, staging or differentiation between fibrotic, benign, malign lesions and inflammation [23–27]. As bone physiologically has low FAPI-uptake, imaging with FAPI-PET/CT is beneficial for evaluation and differentiation of bone lesions such as fibrous dysplasia, rheumatoid arthritis (RA), synovitis, osteitis and OA [21, 28–31]. In RA, FAPI-PET showed high specificity for arthritic joints in vivo and in vitro. Thus, pilot clinical studies suggested FAPI-PET/CT for visualizing upregulated FAP-expression in arthritic synovium [31, 32]. Although FAPI-uptake in joints was described in oncological studies as secondary findings [33, 34], a systematic evaluation of joint-associated ^68^Gallium-labelled- (^68^ Ga) FAPI-signaling with respect to OA and joint degeneration has not been published yet. FAPI-based imaging in musculoskeletal diseases is still in its early stages [35].

In this retrospective analysis, we evaluate the joint-associated FAPI-uptake (FAPI-46, FAPI-74) in PET-imaging of 268 oncological patients. We aimed to quantify joint-associated uptake of both FAPI-variants and to correlate uptake-intensity with radiological severity of joint destruction.

## Materials and methods

### Patient characteristics

To evaluate retrospectively joint-associated ^68^ Ga-FAPI-uptake, we included patients who were referred to ^68^ Ga-FAPI-PET/CT for oncological staging by their treating physicians at the University Hospital of Heidelberg between 04/2018 and 08/2021. Oncological diagnosis, secondary diagnosis, oncological treatment and medication at the time point of PET imaging of each patient were retrospectively extracted from medical records of the hospital information system.

### 68 Ga-FAPI-PET/CT Imaging

Synthesis and labeling of ^68^ Ga-FAPI-46 and ^68^ Ga-FAPI-74 was conducted as previously described [36–38]. For PET imaging, a Siemens Biograph mCT Flow scanner was used, according to previously published protocols [24]. Shortly, after a low-dose CT with or without contrast, 3-dimensional PET-scans were acquired (matrix, 200 × 200), reconstructions performed, and emission data corrected for attenuation. For all patients, static PET-scans were acquired 60 min (min.) post injection (p. i.) of ^68^ Ga-FAPI-46 or ^68^ Ga-FAPI-74. Dependent on the oncological question, 255/268 patients were examined using axial field-of-view, 4 of them including knees, and 13/268 using total-body field-of-view. In 9/255 patients with axial field-of-view the arms were positioned downwards and thus, cubital and thumb joints could be analyzed. In 7/13 patients with total-body field-of-view, these joints could not be analyzed. In 56 patients with FAPI-positive joints (FAPI-46: 33 patients, FAPI-74: 23 patients) supplementary early (15 ± 5 min. p. i.) PET-acquisition was performed.

### Analysis of FAPI-positive joints

^68^ Ga-FAPI-PET-images of all patients were visually analyzed for increased joint-associated uptake by AVG and MR (board-certified nuclear physician). Maximum and mean standardized uptake values (SUVmax/mean) of joint-associated ^68^ Ga-FAPI-uptake and of the bloodpool as background were contoured using a volume of interest (VOI) technique: VOIs of joints were manually drawn according to the individual patient anatomy. VOIs of bloodpool were defined by 8 mm sphere placed into the aortic branch. Target-to-blood ratios (TBRs) were calculated. To objectify visual analysis, only FAPI-positive joints with TBR > 1.2 were included.

### CT-based OA assessment

For CT-based OA-assessment and comparison with joint-associated ^68^ Ga-FAPI-uptake, the acromioclavicular, shoulder and hip joints as most frequent displayed FAPI-uptaking joints in PET/CT were selected. CT-morphology was scored according to the criteria of KL (narrowing of joint cartilage associated with sclerosis of subchondral bone, formation of osteophytes on joint margin or on the tibial spines, small pseudocysts with sclerotic wall in the subchondral bone, altered shape of bone ends, particularly in the femur head and periarticular ossicles, especially in the DIP and PIP-joints) by JH (board-certified radiologist) with grades 0 to 4 (grade 0: no OA, grade 4: most severe level of OA) [16], who was blinded for FAPI-uptake during assessment.

### Laterality analysis of 68 Ga-FAPI-uptake and radiological OA-scores

Laterality of FAPI-uptake was analyzed descriptively with TBRmean > 1.2 on both sides meaning a bilateral FAPI-uptake and TBRmean > 1.2 on the one and ≤ 1.2 on the other side meaning unilateral FAPI-uptake. KL-scores > 0 on both sides were rated as bilateral and KL-scores > 0 on the one and ≤ 0 on the other side as unilateral radiomorphological OA.

### Correspondence of joint associated 68 Ga-FAPI-uptake and radiological OA scores

For PET/CT-correlation, AC, shoulder and hip joints were delineated in PET/CT-data based on CT-morphology in all patients irrespective of visually discernable FAPI-positivity. SUV of these joints and radiological KL-scores were analyzed in view of their consistency.

### Statistical analysis

Statistical analysis was performed descriptively for patients’ characteristics and with chi^2^-test for categorical parameters using Graph Pad Prism 10.2.3. Joint groups were compared applying a linear mixed model with random patient effect to account for multiple joints per patient. For pairwise comparisons of KL-score groups, p-values were adjusted for multiple testing using Tukey’s method. Therefore software R version 4.2 with add-on packages lme4 and lmerTest was used. P-values < 0.05 were considered significant.

### Single cell mass cytometry (cytometry by time of flight, cyTOF)

Mass cytometry data were generated in a multi-center resource study (Accelerating Medicines Partnership® Rheumatoid Arthritis (AMP-RA)) and analyzed as previously described [12, 13]. In brief, single-cell suspensions of synovial tissues from biopsies and arthroplasty surgeries from 15 OA- and 26 RA-patients were incubated with metal ion-linked monoclonal antibodies (n = 35). Data was processed using a manual gating strategy in FlowJo.

### RNA expression analysis

To exclude simultaneous expression of FAP in other cell types than fibroblasts, we queried the online accessible RNA-sequencing dataset from the AMP-RA phase-I dataset (immunogenomics.org, [13]). Using the online function, graphs of bulk and single-cell RNA-sequencing (scRNAseq) were generated displaying RNA-expression profiles from sorted populations of SFs, T-cells, B-cells and monocytes [13]. SFs were defined as being viable, non-doublet, CD45-negative, podoplanin- (PDPN) positive cells.

## Results

### Patient characteristics

We analyzed ^68^ Ga-FAPI-PET/CT scans of 268 patients, (109 women (w), 159 men (m); mean age 59.6 ± 15.1 (w) and 63.3 ± 12.7 (m)). In 159 patients ^68^ Ga-FAPI-46 and in another 109 patients ^68^ Ga-FAPI-74 was applied. supplemental Table 1 provides an overview of patients´ characteristics and supplemental Table 2 of medical history including oncological and rheumatological diagnoses. In 70/268 patients no clinical information beyond oncologic disease was available, e. g. when patients were referred to our hospital exclusively for PET imaging. 3 patients took glucocorticoids at the time point of PET/CT and 3 patients had a documented rheumatic disease in medical history.

### Distribution of visually increased joint-associated 68 Ga-FAPI-uptake

**Fig. 1:**
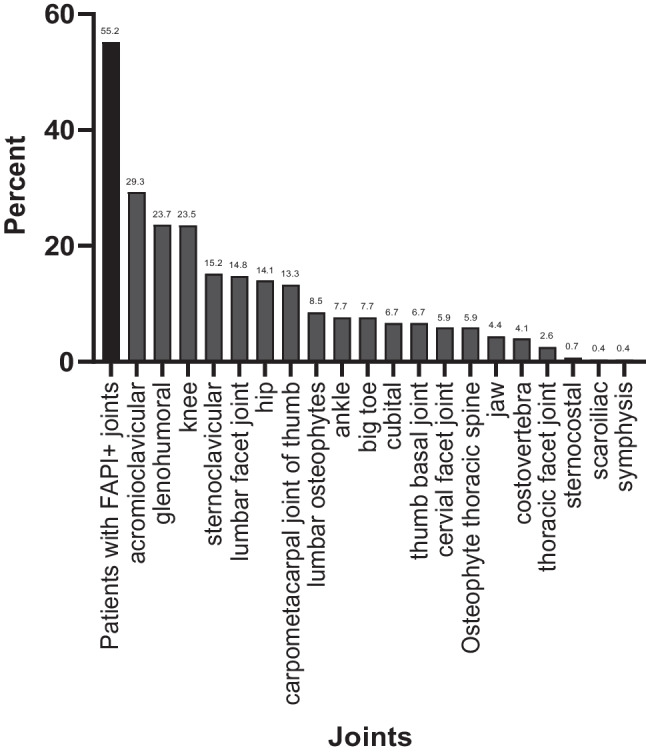
Distribution of fibroblast activation protein inhibitor-46 and −74 (FAPI-46/−74) positive (+) joints in ^68^Gallium-labeled-FAPI positron emission tomography with computed tomography (^68^ Ga-FAPI-PET/CT) in various localizations in 268 oncological patients with 255 axial field-of-view scans (4 including knees) and 13 total-body field-of-view scans

We observed increased joint-associated ^68^ Ga-FAPI-uptake in at least one joint in 55.2% (148/268) of all patients (FAPI-46: 88/159 patients; FAPI-74: 60/109 patients). The frequency FAPI-positivity of individual joints is shown in detail in Fig. 1A. Regarding tracer variants, the portion of patients with ^68^ Ga-FAPI-positive joints was almost identical (FAPI-46 55.35%, FAPI 74: 55.05%).

### Signal-intensities in joints and target-to-blood ratios of FAPI-46 and FAPI-74

**Fig. 2:**
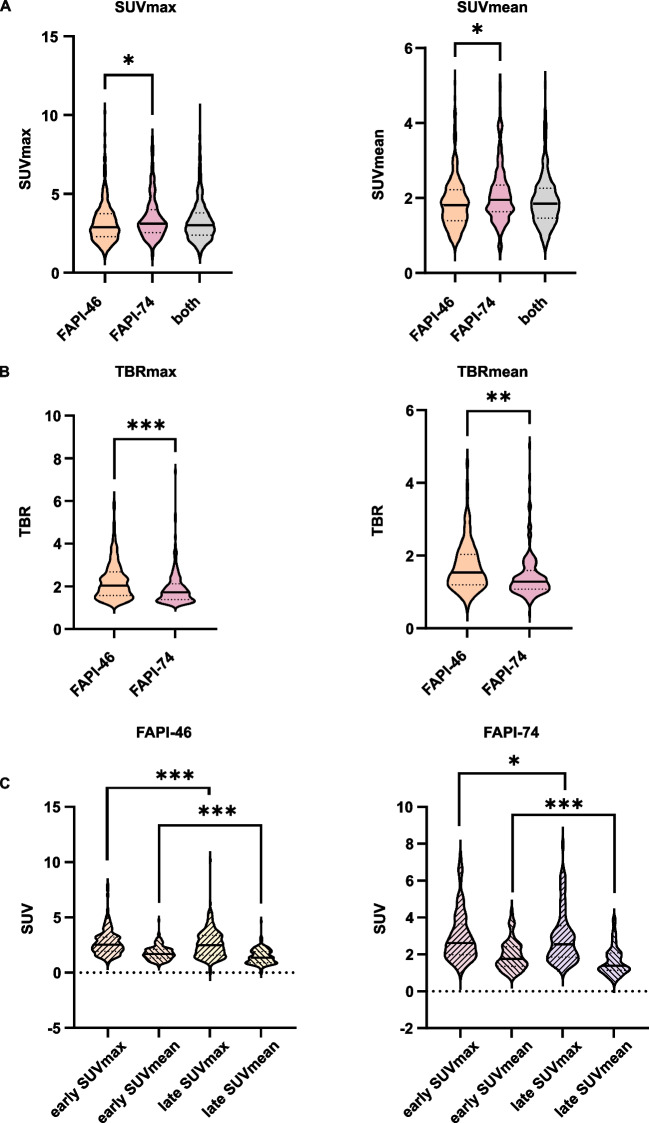
**A** Violin plots of maximum and mean standardized uptake values (SUVmax/mean) of visually ^68^Gallium-labelled fibroblast activation protein inhibitor-46- (^68^ Ga-FAPI-46) positive (378 joints in 159 patients) and FAPI-74-positive joints (183 joints in 109 patients). **B** Violin plots of corresponding maximum and mean tissue to blood ratios (TBRmax/mean). **C** Violin plots of SUVmax/mean of early (15 ± 5 min (min.) and late (60 min.) PET-acquisition time points after administration of ^68^ Ga-FAPI-46 (191 joints in 33 patients) and ^68^ Ga-FAPI-74 (83 (early)/79 (late) joints in 24 patients). The outer shape represents the whole data range, the horizontal line within the box indicates the median and the dotted lines the 1. and 3. quartiles; *, ** and *** mark significant correlations with p-values < 0.05, < 0.01 and < 0.001, respectively

Visually discernable ^68^ Ga-FAPI-positive joints had average SUVmax/mean of 3.25 ± 1.28/1.94 ± 0.70 and TBRs of 2.22/1.64 (TBRmax/mean). For FAPI-46 average SUVmax/mean were 3.15 ± 1.24/1.87 ± 0.68, for FAPI-74 3.45 ± 1.34/2.07 ± 0.72, respectively (Fig. 2A). The average TBR of FAPI-46-positive joints was significantly higher (SUVmax/mean 2.25 ± 0.89/1.70 ± 0.68) than of FAPI-74-positive joints (SUVmax/mean 1.91 ± 0.80/1.45 ± 0.62) (Fig. 2B). Regarding different acquisition time points, early imaging showed average SUVmax/mean of 2.83 ± 1.35/1.72 ± 0.67 (FAPI-46) and 2.85 ± 1.32/1.75 ± 0.66 (FAPI-74). Average SUVmax/mean for late imaging (60 min p. i.) were 2.59 ± 1.42/1.50 ± 0.68 (FAPI-46) and 2.59 ± 1.42/1.49 ± 0.68 (FAPI-74) (Fig. 2C). With regard to different joint type, the highest average FAPI-uptake was observed in shoulder (SUVmax/mean 3.63 ± 1.33/1.95 ± 0.68) and hip joints (SUVmax/mean 4.06 ± 1.83/2.25 ± 1.0). The lowest average uptake was observed in sacroiliac joints (SUVmax/mean 2.18 ± 0.18/1.44 ± 0.03). supplemental Table 2 provides an overview of signal intensities in joints.

### Laterality analyses of 68 Ga-FAPI-uptake and radiological OA-scores

**Fig. 3:**
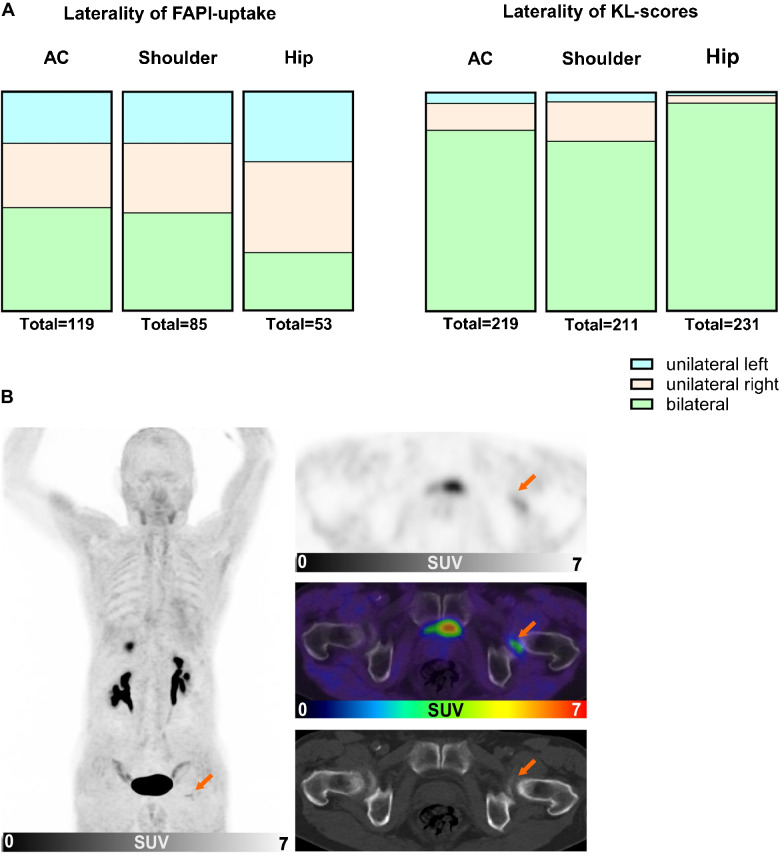
**A** Laterality of the fibroblast activation protein inhibitor- (FAPI) uptake (TBRmean > 1.2) and of the Kellgren and Lawrence (KL) scores in acromioclavicular (AC), shoulder and hip joints of 356 patients. The distribution of radiomorphological osteoarthritis is almost always symmetric (78–95% of cases), while FAPI-uptake is in 53–74% of cases asymmetric. **B** Representative images of ^68^Gallium-labelled-FAPI positron emission tomography with computed tomography (.^68^ Ga-FAPI-PET/CT) and CT of an exemplary hip joint from a patient with a symmetric KL-score (KLS grade 2) and an asymmetric maximum standardized uptake values (SUVmax: left 2.1, right 0.9)

119 AC, 85 shoulder and 53 FAPI-positive hip joint pairs were included into laterality analysis. In AC joints, we detected FAPI-positivity in 23.5% unilateral left, in 29.4% unilateral right and in 47.0% bilateral; in shoulder joints in 23.5% unilateral left, in 31.8% unilateral right and in 44.7% bilateral; in hip joints 32.1% unilateral left, in 41.5% unilateral right and in 26.4% bilateral (Fig. 3A). We also investigated the laterality of OA by determining bilateral KL-scores. For laterality of KL-scores, we analyzed 219 AC, 211 shoulder and 231 hip joint pairs and compared the frequency of identical KL-scores in the joint pairs per patient. KL-scores > 0 were for AC joints 5.0% unilateral left, 12.3% unilateral right and 82.7% bilateral; for shoulder joints 4.3% unilateral left, 18.1% unilateral right and 77.3% bilateral; for hip joints 1.7% unilateral left, 3.5% unilateral right and 94.8% bilateral (Fig. 3B). Taken together, FAPI-positivity in joints was asymmetric in the majority (53–74%) of cases, while the distribution of OA was symmetric in 78–95% of cases.

### Comparison of joint-associated 68 Ga-FAPI-uptake and radiological OA-scores

**Fig. 4:**
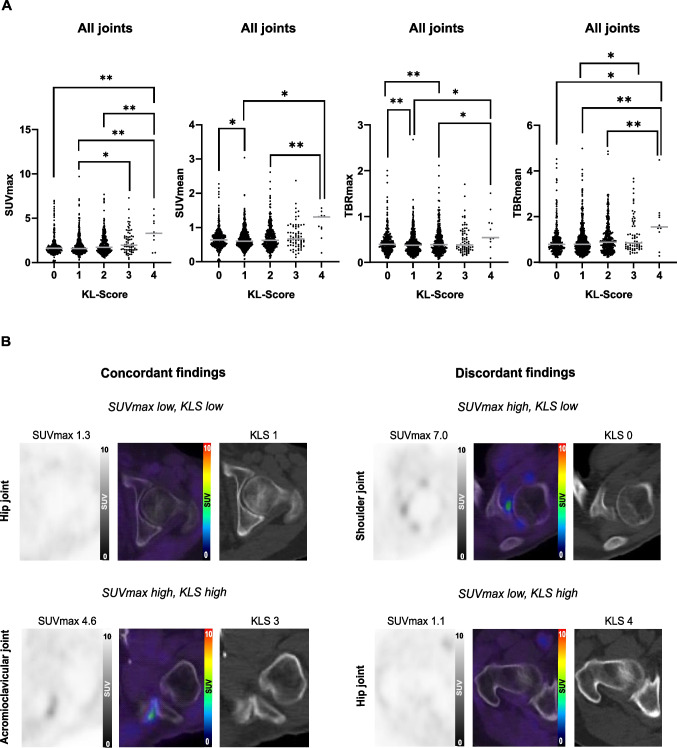
**A** Maximum and mean standardized uptake values (SUVmax/mean) and tumor to blood rations (TBRmax/mean) in 1457 joints (including acromioclavicular (AC), shoulder and hip joints) depending on Kellgren and Lawrence-scores (KLS). The grey horizontal line indicates the median; * and ** mark significant correlations with p-values < 0.05 and < 0.01, respectively. **B** Representative images of ^68^Gallium-labelled-FAPI positron emission tomography with computed tomography (^68^ Ga-FAPI-PET/CT) showing either concordance or discordance of KLS and SUVmax in AC, shoulder or hip joints

In order to test for a relation between FAPI-uptake and presence of OA and its severity, we compared measures of FAPI-uptake to corresponding KL-scores in individual patients in the total cohort. In 19 patients, not all data of the three selected joint types (AC, shoulder, hip) could be used for the statistical analysis due to overall reduced CT quality (3 patients), shoulder total endoprothesis (TEP) (2 patients), hip TEP (12 patients) and metastasis in hip bone (2 patients). In total, AC joints of 265 patients, shoulder joints of 263 patients and hip joints of 251 patients were finally analyzed. When pooling all joints from all included patients, we observed 356 joints with KL-score "0", 890 joints with KL-score "1", 586 joints with KL-score "2", 168 joints with KL-score "3" and 83 joints with KL-score "4". Statistical analyses revealed that there were significant differences in SUVmax depending on KL-scores, as shown in Fig. 4A, left graph. Also, for SUVmean, TBRmax and TBRmean, significant differences were observed depending on KL-scores (Fig. 4A). These data indicate a tendency towards higher FAPI-uptake in higher degraded joints; however, FAPI-uptake was heterogeneous in each KL-score group and the ranges largely overlapped, indicating a rather loose relationship between FAPI-uptake and OA severity. Given this loose relationship between FAPI-uptake and KL-scores, we screened for interfering clinical characteristics (supplemental Table 2). Hypothesizing that synovial FAP-expression might depend on inflammatory signals [39], we evaluated the available data on glucocorticoid intake in our cohort. Based on the retrospectively available clinical information from the patient records, three patients were identified, who were taking glucocorticoid at the time of FAPI-PET/CT. The median SUVmax/mean in 18 joints of these 3 patients with glucocorticoids and KL-score “0” (2.1/0.8), KL-score “1” (2.3/0.7) and KL-score “2” (1.3/0.5) did not clearly differ from the whole patient cohort (data not shown). There were no joints with KL-score “3” or “4” in these patients.

The statistical distribution of FAPI-uptake in the different KL-score groups indicated that even severe joint damage (KL-score "3" or "4") can be associated with low FAPI-uptake. Vice versa, high FAPI-uptake can also be found in joints without degeneration (KL-score "0"). An analysis of KL-scores within the group of joints that were FAPI-positive confirmed that joints with no radiologically visible OA (KL-score “0") presented similar mean FAPI-uptake as joints with signs of OA (KL-score “1” and “2”; supplemental Fig. 1). Exemplary cases of concordance and discordance of FAPI-uptake and joint degeneration are displayed in Fig. 4B. A jointwise (AC, shoulder, hip) comparison of SUVmax/mean with KL-scores is shown in supplemental Figs. 2 , 3 shows jointwise (AC, shoulder, hip) images of concordant and discordant findings.

### Validation of surface FAP-expression on fibroblasts in synovial tissue in OA

**Fig. 5:**
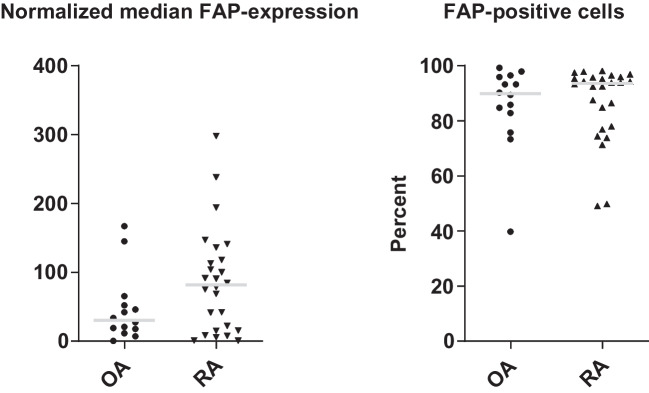
Single cell cytometry by time of flight (CyTOF) analysis in a different cohort with high-stage osteoarthritis (OA) and rheumatoid arthritis (RA). Normalized median expression and percentage of fibroblast activation protein (FAP)-positive fibroblasts in OA and RA joints are shown, indicating similar levels of median FAP-expression (left) and FAP-positive fibroblasts (right). The CyTOF raw data were generated in a multi-center resource study (Accelerating Medicines Partnership® Rheumatoid Arthritis (AMP-RA) and analyzed as previously described [12, 13]

As described above, a significantly higher FAPI-uptake was observed in patients with the highest KL-score "4". As this result is the strongest support of an association of (severe) OA with FAPI-uptake in our study, we validated the expression of FAP in severe OA undergoing surgery for joint replacement, using a proteomics approach. To this end, we analyzed mass cytometry (cyTOF) raw-data derived from synovial tissue from OA and RA from a different patient cohort as described earlier [12, 13]. As most of the patients in this dataset donated synovial tissue during joint replacement surgeries, the cohort can be regarded as late stage OA and RA. Both median expression and the percentage of FAP-positive fibroblasts were indifferent between OA and RA, with only a tendency of more FAP-expression in RA (Fig. 5). In bulk-RNA-sequencing we found that RNA-expression of FAP was restricted to fibroblasts both in OA and RA compared to B-cells, T-cells and monocytes (supplemental Fig. 3A). Additionally, scRNAseq showed that FAP was distributed on all fibroblast clusters (supplemental Fig. 3B). Together, FAP is expressed on SFs in higher-stage OA.

## Discussion

The key finding of our retrospective study of 268 patients is the high frequency of FAPI-positivity in joints in more than 50% of patients. This frequent occurrence prompted us to investigate its relationship to the most frequent joint disease, OA. Our first approach, the laterality analyses of FAPI-positivity and OA-scores revealed a pronounced unilaterality of FAPI-uptake. This finding argues against a tight relationship to OA, which was in most cases symmetric. In line, the degree of FAPI-uptake was overlapping between the different levels of OA-scores, even though statistical analysis indicated a more pronounced FAPI-uptake in higher-stage OA. Finally, FAPI-PET/CT revealed FAP-expression also in joints without any signs of radiological joint destruction. While these data overall suggest that FAPI-uptake can be independent of existing joint destruction, it seems that high-grade OA is more likely to be associated with higher FAPI-uptake. The latter can be regarded as confirmed by FAP-expression in joints as determined by our single-cell-analyses. Nevertheless, degenerated joints with KL-scores of 2, 3 and 4 might also come along with low FAPI-uptake. In these joints, FAPI-uptake is absent despite signs of joint destruction. While necessitating future targeted studies for clarification, several factors might account for low FAPI-uptake in destructed joints: OA is increasingly regarded as a heterogeneous condition in which diverging mechanisms lead to destruction; inflammatory OA-forms are distinguished from rather fibrotic forms [40]. Alternatively, there might be a temporal component in which destructions might be caused by repetitive phases of synovial/fibroblast activity which are interrupted by quiescent phases. In addition, non-investigated factors such as pain, immobility or pre-investigation exercise as well as technical limitations might also contribute to discordant findings of FAPI-uptake and joint destructions.

While our study is the first to systematically compare FAPI-uptake with joint-degeneration, the results of our analyses expand previous knowledge on fibroblast activation and FAPI-uptake in joints [27, 41, 42]. One study supports our result that AC joints are relatively frequently affected by non-malignant FAPI-uptake [43]. Other studies compared the uptake of ^68^ Ga-FAPI to ^18^F-FDG in RA; ^68^ Ga-FAPI showed higher detection rate of affected joints and higher SUVmax. Additionally, FAPI correlated with RA-activity [27] suggesting inflammation as a driver of FAPI-uptake. The connection between FAPI-uptake and inflammatory activity was confirmed in mouse models of RA and OA. Furthermore, FAPI-uptake was observed in RA in inflamed joints before joint deformity could be proven histologically [41]. A similar observation was made in lung fibrosis where intensive FAPI-positive lesions correlated with fibrotic activity and thus clinical progression [44]. SUVmax/mean of pulmonary lobes correlated with CT-based indices for lung fibrosis [24]. Further expanding the potential role of fibroblasts in these diseases, we have previously described FAPI-uptake and thus fibroblast pathology despite clinical long-term remission, suggesting that persisting fibroblast activation is a driver of chronicity and relapses in aortitis [22]. Thus, the present study underpins a potential use of FAPI-PET/CTs in musculoskeletal and soft tissue remodeling beyond cancer.

A disadvantage of ^18^F-FDG in context of PET-imaging in patients with OA and RA might be the influenceability by glucocorticoid therapy via its impact on glucose metabolism [45]. FAPI-imaging, relying on a fibroblast-specific molecule, is not dependent on glucose metabolism and could be ideal for detecting and quantifying fibroblast pathology, potentially aiding in the monitoring of therapies targeting fibroblasts in diseases like OA, RA, fibrosis, and aortitis [46–48].

If FAP and/or persisting fibroblast activation is indeed an early event or even driver of OA and joint-degeneration—which may also be an interpretation of our study results—a fibroblast-targeting strategy may reveal successful to treat or prevent OA. At the same time, our study shows that care has to be taken when activated fibroblasts are targeted by new treatment strategies, given their frequent presence in yet to be more clearly defined joint scenarios.

Our analysis showed no marked difference in the frequency or intensity of FAPI-uptake in FAPI-46 and FAPI-74. There are a few smaller studies which analyzed FAPI-uptake in joints using just one FAPI-compound [27, 31, 49, 50]. The first structured comparison of FAPI-02, −46 and −74 uptake in malignant, inflammatory/reactive (IR) and degenerative lesions was performed by Glatting et al. (2022). They showed higher uptake in IR than in degenerative lesions for FAPI-46 and −74 with more pronounced differences for FAPI-46 than for FAPI-74. FAPI-02 showed similar SUV in IR and degenerative lesions. Regarding biodistribution and TBR, FAPI-46 showed the highest uptake by muscle but the highest TBR for all types of pathologies [51]. In conclusion, FAPI-46 might be theFAPI-compound of choice for a precise differentiation between IR and degenerative lesions such as OA. FAPI-74 would be good alternative in centers with limited availability of ^68^ Ga or in situations where the short half-life of ^68^ Ga is limiting as labeling with ^18^F is also possible [52]. However, it should be taken under consideration that this study is the first to analyze FAPI-74-uptake in OA-joints and to date no study has specifically investigated FAPI-74 in RA- or OA-joints.

Our findings, in line with emerging literature, show that FAPI-uptake in joints is a frequent phenomenon. These results may have implications for the clinical practice for both nuclear medicine physicians and clinicians. As we showed that every second oncological patient can show FAPI-uptake in joints with or without association to radiological OA-signs, it is important to interpret joint-associated FAPI-uptake with caution in context of oncologic imaging. Especially focal FAPI-uptake in the immediate vicinity of the bone can potentially lead to the suspicion of bone metastasis, in particular when accompanied by morphological signs of bone changes. Such potential pitfall can impact oncological treatment decisions. It is therefore important to recognize that FAPI-uptake in joints frequently occurs independent of tumors. Similarly, FAPI-uptake being suggested as a new potential measure of disease activity in RA [27], our study results favor a careful interpretation of FAPI-uptake in joints.

A number of limitation of our study must be considered. Firstly, the inclusion of two different FAPI-compounds in our analysis may bias the results. With regard to the quantitative analysis, we detected no major differences in SUV and TBR between FAPI-46 and −74 in this cohort. With regard to the detection of inflammatory/reactive or degenerative changes comparable detection rates for both compounds were demonstrated by our group [51]. Secondly, no statement could be made about knees due to cancer-directed patient positioning (elevated arms, knees mostly not included). The low number of patients with KL-score of “4” somewhat limits the interpretation that high FAPI-uptake occurs in particular in end-stage OA, even though FAPI-uptake in joints with a KL-score of “4” showed the most statistical significances compared to other KL-scores. Regardless of this, the expression of FAP in high-stage OA was confirmed using CyTOF. The retrospective character is another main limitation of our study. Medication, in particular immunosuppression and chemotherapy, could potentially be an interfering factor. Of note, there was only a small number of patients under glucocorticoid therapy and these patients showed a comparable FAPI-uptake in relation to KL-score as the total patient cohort. In most cases, other immunosuppressants or chemotherapies at the time of the FAPI-PET/CT were not documented with satisfying certainty and could not be evaluated. We could not account for pain or laboratory at the time point of PET/CT and thus could not correlate FAPI-uptake with patients' symptoms. To overcome these limitations, future prospective studies are needed including patient-reported symptoms such as a standardized pain scales, joint-specific history and medication.

## Conclusion

Our study, along with current literature, indicates that FAPI-uptake is associated with various joint and stromal diseases, probably preceding radiologically measurable tissue damage. Whether FAPI-uptake in morphologically healthy joints indicates ongoing pathophysiological processes leading to degeneration requires further investigation. The triggers of FAPI-uptake in normal joints remain speculative, potentially including subclinical injuries, tissue repair, pain, exercise or posture.

## Supplementary Information

Below is the link to the electronic supplementary material.

Supplementary file1 (DOCX 834 KB)

## Data Availability

The data that support the findings of this study are not openly available due to reasons of sensitivity and are available from the corresponding author upon reasonable request. Data are located in controlled access data storage at University Medical Center Mainz.
