# Supplementary material for: 68Gallium-labelled fibroblast activation protein inhibitor uptake in joints: a single-center cohort analysis of 268 patients
Source: Eur J Nucl Med Mol Imaging. 2025 Nov 20;53(5):3226–37. doi: 10.1007/s00259-025-07636-x (PMC13013341; doi:10.1007/s00259-025-07636-x)
Supplement: Supplementary file 1 — Supplementary file1 (DOCX 834 KB) [file 259_2025_7636_MOESM1_ESM.docx]

**Supplemental material**

**^68^Gallium-labelled fibroblast activation protein inhibitor uptake in joints: a single-center cohort analysis of 268 patients**

Anna-Maria Spektor^1^, Antonia van Genabith^2^, Jorge Hoppner^2^, Leon Walkenbach^2^, Thomas Hielscher^3^, Peter Kvacskay^4^, Sarah Richter^4^, Kiangenda Trésor Sungu-Winkler^4^, Hans-Ulrich Kauczor^5,6^, Hanns-Martin Lorenz^4^, Mathias Schreckenberger^1^, Jörg Distler^7^, Uwe Haberkorn^2,6,8^, Wolfgang Merkt^4,7*^ and Manuel Röhrich^1*^

**Affiliations:**

1. Department of Nuclear Medicine, University Hospital Mainz, Germany.
2. Department of Nuclear Medicine, University Hospital Heidelberg, Germany
3. Department of Biostatistics, German Cancer Research Center, Heidelberg, Germany.
4. Department of Internal Medicine V Hematology Oncology Rheumatology, University Hospital Heidelberg, Germany
5. Department of Diagnostic and Interventional Radiology, University Hospital Heidelberg, Germany.
6. Translational Lung Research Center Heidelberg (TLRC), Member of the German Center for Lung Research DZL, Heidelberg, Germany.
7. Department of Rheumatology, University Hospital Düsseldorf, Germany
8. Clinical Cooperation Unit Nuclear Medicine, German Cancer Research Center (DKFZ), Heidelberg, Germany.

*shared last authors

**Corresponding author:**

Wolfgang Merkt

Moorenstr. 5

40225 Düsseldorf

Telephone: +49-211-811-7817

Fax number: +49-211-811-6455

Email: wolfgang.merkt@med.uni-duesseldorf.de

ORCID-ID: 0000-0002-3108-154X

**First author:**

Anna-Maria Spektor

Langenbeckstraße 1

55131 Mainz

Email: anna-maria.spektor@unimedizin-mainz.de

ORCID-ID: 0009-0005-1092-5773

**Supplemental table 1:** Patients’ characteristics

| **FAPI-variant** | **Cases** | **Women** | **Age** | **Men** | **Age** | **Type of cancer** | | | |
| --- | --- | --- | --- | --- | --- | --- | --- | --- | --- |
|  |  |  |  |  |  | **PDAC** | **NSCLC** | **GIC** | **Other** |
| both | 268 | 109 | 59.6 ± 15.1 | 159 | 63.3 ± 12.7 | 71 | 32 | 49 | 116 |
| 46 | 159 | 67 | 58.7 ± 16.7 | 92 | 62.8 ± 13.5 | 47 | 17 | 25 | 70 |
| 74 | 109 | 42 | 61.1 ± 12.3 | 67 | 64.0 ± 11.5 | 24 | 15 | 24 | 46 |
| Abbreviations: FAPI: Fibroblast activation protein inhibitor; PDAC: Pancreatic ductal adenocarcinoma; NSCLC: Non-small cell lung cancer; GIC: Gastrointestinal cancer | | | | | | | | | |

**Supplemental table 2:**  Medication and rheumatic secondary diagnosis

| **Patient number** | **Medication** | | | **Rheumatic disease** | | |
| --- | --- | --- | --- | --- | --- | --- |
|  | **Immunotherapy** | **Glucocorticoids** | **NSAID** | **RA*** | **Vasculitis** | **Sjögren´s Syndrome** |
| 268 | 0 | 3 | ND | 6 | 2 | 1 |
| Abbreviations: NSAID: Nonsteroidal anti-inflammatory drugs; RA: Rheumatoid arthritis; ND: not documented; *location not further specified | | | | | | |

**Supplemental table 3:** Visually assessed maximum and mean standardized uptake values (SUVmax/mean) of joint-associated FAPI-uptake in ^68^Ga-FAPI-46 and -74-PET/CT of 268 oncological patients

| **Joints** | **SUVmax** | **SUVmean** | **Number of patients** | **Number of joints** |
| --- | --- | --- | --- | --- |
| Acromioclavicular joint | 3.12 ± 1.04 | 1.95 ± 0.64 | 79 | 127 |
| Shoulder joint | 3.63 ± 1.33 | 1.95 ± 0.68 | 64 | 109 |
| Sternoclavicular joint | 3.17± 1.09 | 1.93 ± 0.62 | 41 | 65 |
| Lumbar facet joint | 3.13 ± 1.04 | 1.95 ± 0.58 | 40 | 63 |
| Hip joint | 4.06 ± 1.83 | 2.25 ± 1.00 | 38 | 63 |
| Osteophytes of lumbar vertebral bodies | 2.56 ± 0.87 | 1.56 ± 0.50 | 23 | 25 |
| Cervical facet joint | 3.24 ± 1.12 | 2.16 ± 0.70 | 16 | 24 |
| Osteophytes of thoracic vertebral bodies | 2.53 ± 0.62 | 1.61 ± 0.39 | 16 | 16 |
| Mandibular joint | 2.47 ± 1.24 | 1.63 ± 0.77 | 16 | 16 |
| Costovertebral joint | 2.92 ± 0.89 | 1.91 ± 0.48 | 11 | 15 |
| Thoracic facet joint | 2.67 ± 0.66 | 1.75 ± 0.44 | 7 | 13 |
| Knee joint | 2.40 ± 0.65 | 1.30 ± 0.41 | 4 | 6 |
| Carpometacarpal joint of the thumb | 2.83 ± 0.52 | 1.82 ± 0.32 | 2 | 4 |
| Sternocostal joint | 3.11 ± 1.68 | 1.80 ± 1.33 | 2 | 2 |
| Ankle joint | 4.62 ± 2.90 | 2.47 ± 1.65 | 1 | 2 |
| Big toe joint | 2.22 ± 0.09 | 1.35 ± 0.09 | 1 | 2 |
| Sacroiliac joint | 2.18 ± 0.18 | 1.44 ± 0.03 | 1 | 2 |
| Thumb basal joint | 2.56 ± 0.00 | 1.82 ± 0.00 | 1 | 1 |
| Cubital joint | 2.54 ± 0.00 | 1.50 ± 0.00 | 1 | 1 |
| Symphysis | 8.01 ± 0.00 | 4.27 ± 0.00 | 1 | 1 |

**Supplemental figure 1**

**
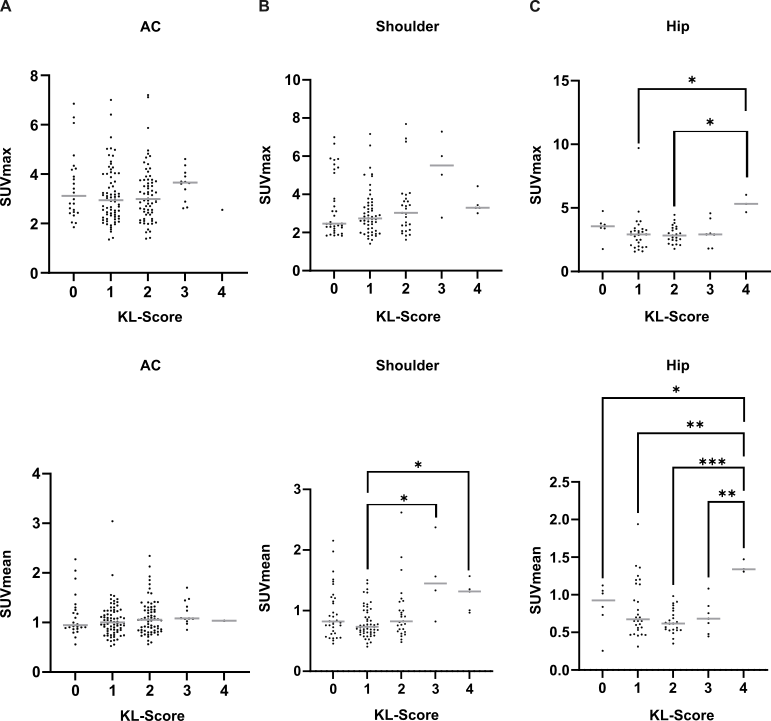
**

**A, B, C** Comparison of SUV of FAPI-positive (defined by mean tissue-to-background ratio (TBRmean) > 1.2) acromioclavicular (AC; **A**), shoulder (**B**) and hip (**C**) joints with Kellgren and Lawrence (KL) scores. Joints without radiomorphological signs of osteoarthritis (OA) (KL score 0) show similar high uptake of fibroblast activation inhibitor (FAPI) like joints with low or medium (KL scores 1 and 2) severity level of OA. The grey horizontal line indicates the median; *, ** and *** mark p-values < 0.05, < 0.01 and < 0.001, respectively.

**Supplemental figure 2**

**
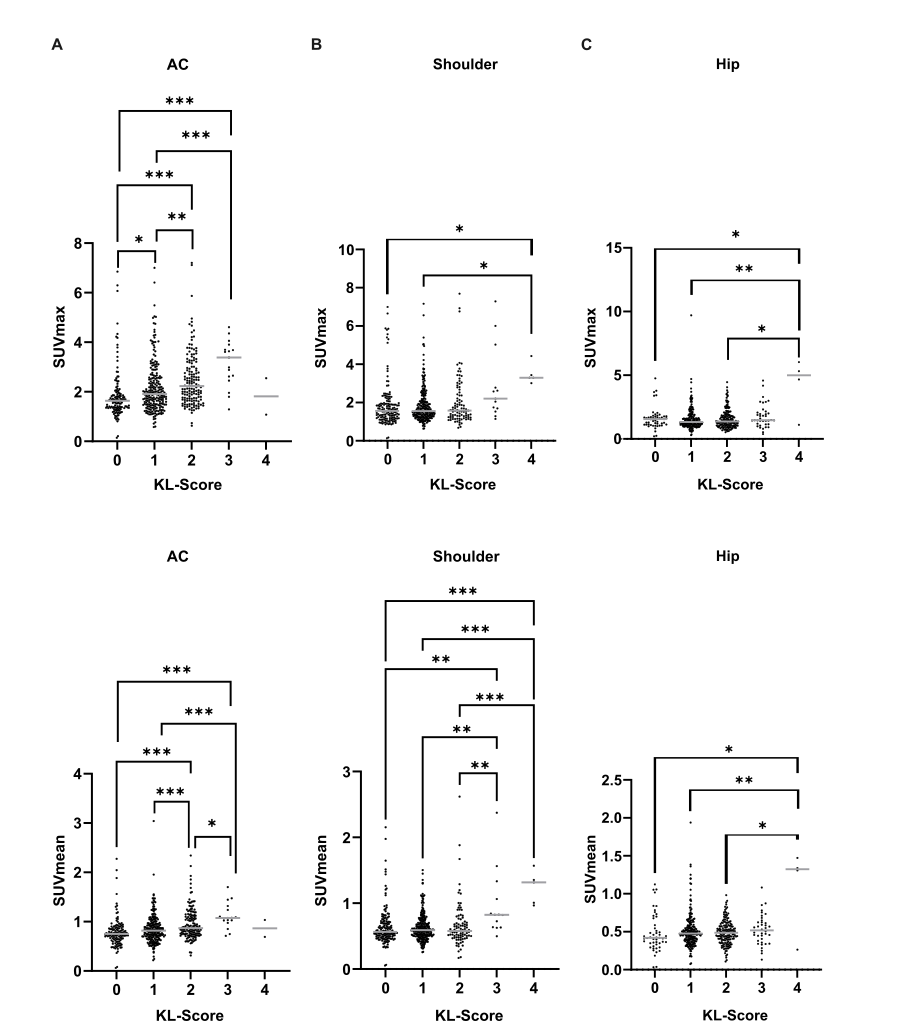
**

**A, B, C** Comparison of SUV with Kellgren and Lawrence (KL) scores . SUVmax/mean of all acromioclaviucular (AC) joints (530 joints) (**A**), SUVmax/mean of all shoulder joints (426 joints) (**B**), SUVmax/mean of all hip joints (501 joints) (**C**). The grey horizontal line indicates the median; *, ** and *** mark p-values < 0.05, < 0.01 and < 0.001, respectively. There is no tight concordance between SUVmax, SUVmean and KL-scores.

**Supplemental figure 3**


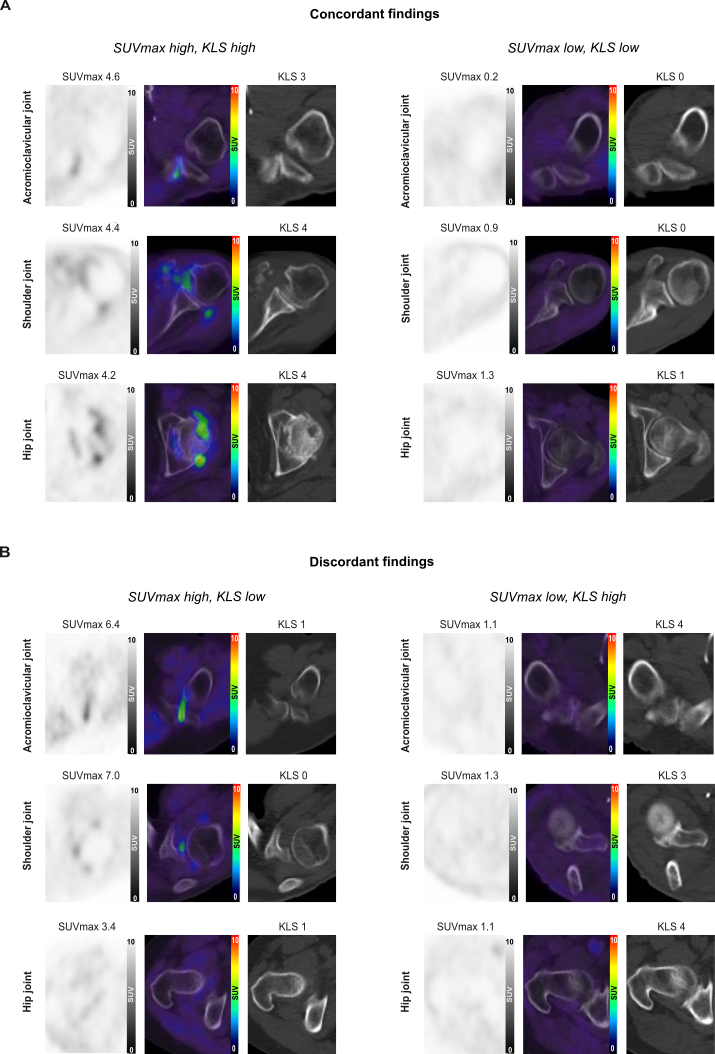


**A, B** Representative images of ^68^Gallium-labelled-FAPI positron emission tomography with computed tomography (^68^Ga-FAPI-PET/CT) showing either concordant (A) or discordant (B) findings of Kellgren and Lawrence-scores (KLS) and SUVmax in acromioclavicular, shoulder and hip joints.


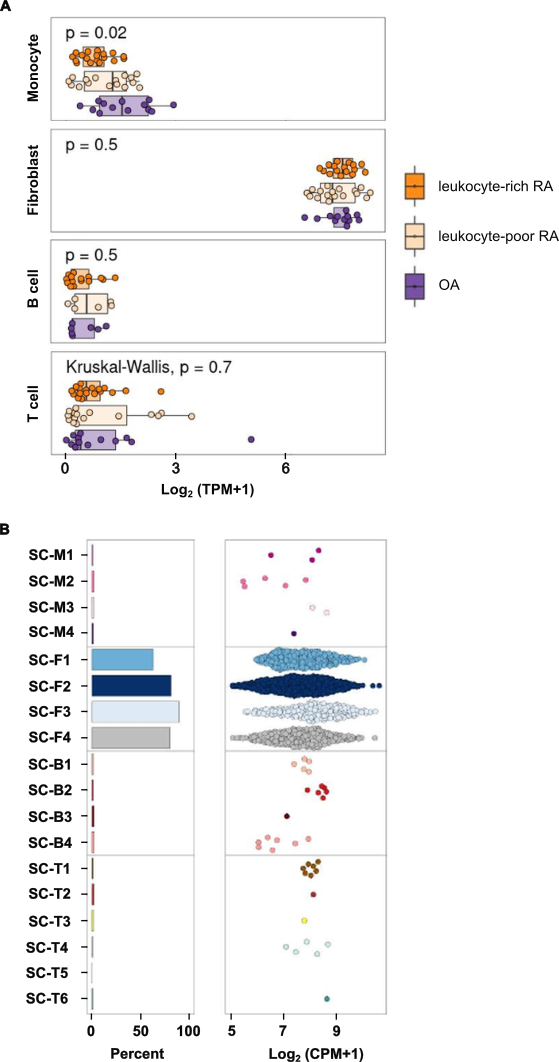
**Supplemental figure 4**

**RNA-expression analysis in various synovial cell types** **in another patient collective**. Synovial tissue derived from knee joint replacement surgery in patients with osteoarthritis (OA)- and rheumatoid arthritis (RA) was analyzed by single cell RNA sequencing as previously described (immunogenomics.org, [13]).Fibroblasts are the major synovial cell population with FAP-expression, as compared to B-cells, T-cells and monocytes. **A** bulk RNA sequencing**. B** single cell RNA sequencing. For each cell type, four to six clusters are depicted, confirming FAP expression in synovial fibroblasts (immunogenomics.org, [13]).
